# Entrapment of Acridine Orange in Metakaolin-Based Geopolymer: A Feasibility Study

**DOI:** 10.3390/polym15030675

**Published:** 2023-01-28

**Authors:** Antonio D’Angelo, Luigi Vertuccio, Cristina Leonelli, Mohammad I. M. Alzeer, Michelina Catauro

**Affiliations:** 1 Department of Engineering, University of Campania “Luigi Vanvitelli”, Via Roma n. 29, 81031 Aversa, Italy; 2 Department of Environmental, Biological and Pharmaceutical Sciences and Technologies, University of Campania “Luigi Vanvitelli”, Via Vivaldi 43, 81100 Caserta, Italy; 3 Department of Engineering “Enzo Ferrari”, University of Modena and Reggio Emilia, Via P. Vivarelli 10, 41125 Modena, Italy; 4 Fibre and Particle Engineering Research Unit, University of Oulu, Pentti Kaiteran Katu 1, 90014 Oulu, Finland

**Keywords:** geopolymers, acridine orange, UV-Vis, FT-IR, flexural and compressive strength

## Abstract

Few studies have explored the immobilization of organic macromolecules within the geopolymer matrix, and some have found their chemical instability in the highly alkaline geopolymerization media. The present work reports on the feasibility of encapsulating the potentially toxic acridine orange (AO) dye in a metakaolin based geopolymer while maintaining its structural integrity. The proper structural, chemical, and mechanical stabilities of the final products were ascertained using Fourier-transform infrared (FT-IR) spectroscopy, scanning electron microscopy (SEM), X-ray diffraction (XRD), thermogravimetric (TGA/DTG), and mechanical analyses, whereas the dye integrity and its stability inside the geopolymer were investigated by the UV-Vis analysis. In addition, the antimicrobial activity was investigated. The FT-IR and XRD analyses confirmed the geopolymerization occurrence, whereas the TGA/DTG and mechanical (compressive and flexural) strength revealed that the addition of 0.31% (AO mg/ sodium silicate L) of AO to the fresh paste did not affect the thermal stability and the mechanical properties (above 6 MPa in flexural strength and above 20 MPa for compressive strength) of the hardened product. UV-Vis spectroscopy revealed that the dye did not undergo chemical degradation nor was it released from the geopolymer matrix. The results reported herein provide a useful approach for the safe removal of toxic macromolecules by means of encapsulation within the geopolymer matrix.

## 1. Introduction

Metakaolin, MK (Al_2_Si_2_O_7_), the product of thermal dehydroxylation of kaolinite (Al_2_Si_2_O_5_(OH)_4_), has been widely used as a model aluminosilicate precursor in the evolution of alkali-activated solids [1,2,3]. The extremely fine grain size of such a mineral assures an extended dissolution of Al^3+^ and Si^4+^ ions in strong alkaline media where they generate hydrated moieties, Al(OH)_4_^−1^ and Si(OH)_4_ [4]. Once these OH-rich species condense in a gel, the expulsion of water molecules can occur at room temperature soon after or concurrently with dissolution. Over time, the more thermodynamically stable structure of the aluminosilicate 3D network takes over, leading to a solid material that retains the crystalline species that were not dissolved, typically alpha-quartz traces present in the pristine kaolinite [5]. The final solidified materials, also known as geopolymers [6], have exceptional chemical, thermal, and mechanical properties if compared with room temperature solidified binders based on lime, gypsum, and clinker. 

One of the peculiar chemical properties of these geopolymers is the capability to encapsulate additional cations and anions through the stabilization/solidification process [7,8] and even macromolecules [9,10,11,12] remaining entrapped during the aluminosilicate 3D network, which presents crypto-zeolite local organization [13]. Because of the presence of zeolite A in the formulations of geopolymers based on Brazilian metakaolins [14], the consolidated geopolymers displayed high adsorption capacity within short times (between one and five minutes) for different cationic dyes, i.e., methylene blue, safranin, and malachite green from aqueous solutions. From the observation of such an encapsulating capability, the number of studies dedicated to different cations [15,16,17,18,19], anions [20,21], and larger molecular assemblages [22] has increased over the last few years. 

Recent years have witnessed a particular interest in the encapsulation of organic dyes within geopolymer pastes. Typically, organic dyes possess insufficient chemical stability at pH = 14, at which the first step of geopolymerization (i.e., the dissolution) occurs. In fact, most of organic dyes undergo decomposition at such a high pH. However, pH indicator molecules represent an exception in which they retain their structure and color in high alkaline media [22] allowing their encapsulation within the geopolymer framework. In this context, the incorporation of various dyes such as methylene blue, crystal violet, acid blue, and Congo red in an MK-based geopolymer has recently been shown to be viable [23].

In this study, we report on the encapsulation of the potentially toxic acridine orange (AO) dye into a freshly prepared geopolymer paste. Acridine orange is a synthetic organic dye with a planar heterocyclic structure, and it is used in several fields such as printing, dyeing textiles and leather, and lithography [24]. The AO is also used as a probe for the investigation of the cellular cycle. Indeed, it changes its fluorescent emission properties with biological polymers such as DNA or RNA [25]. Because of its ability to interact with DNA and RNA, it has potential mutagenic and carcinogenic effects [26]. As with other toxic industrial dyes, their removal from the environment is of great interest [27,28,29]. Hence, its disposal can be substituted by a cleaner encapsulation process.

The main objective of this paper is to investigate the feasibility of entrapping acridine orange in the three-dimensional structure of the geopolymer without the degradation of the molecule nor its release from the geopolymer matrix, thus obtaining materials with possible applications for waste removal as well as for restoration. In particular, in this preliminary study, acridine orange powder was previously dissolved in ethanol (because of its high solubility in this solvent) and then added to the fresh geopolymer paste during the mixing procedure. The inertization process via alkaline activation of metakaolin with soda and sodium silicate solutions was ascertained by microstructural analyses (FT-IR, SEM, TG/DTA, and XRD) and by leaching tests in water. Moreover, the mechanical behavior of the synthesized geopolymers has also been investigated to confirm the structural stability of the 3D aluminosilicate network of the consolidated products.

## 2. Materials and Methods

### 2.1. Materials

The used aluminosilicate source was a commercial white metakaolin purchased from IMCD Deutschland GmbH & Co. (Köln, Germany) (d_50_ = 3.6 µm, surface area via B.E.T. method 12 m^2^/g), whose chemical composition is presented in Table 1.

The metakaolin was activated by using sodium hydroxide (Sigma-Aldrich, Darmstadt, Germany) and sodium silicate (Prochin Italia Prodotti Chimici Industriali Srl, Marcianise, Italy) (chemical composition is presented in Table 1), whereas acridine orange base, 3,6-bis(dimethylamino)acridine supplied by Sigma-Aldrich, Darmstadt, Germany, was added in the form of powder dissolved in ethanol. All the reagents used for the analyses were of analytical grade. 

### 2.2. Geopolymer Synthesis

The comparative study of geopolymers with and without the addition of 0.31 wt% dye on a wet paste basis was carried out by keeping all the parameters of the mixtures constant, i.e., liquid/solid ratio, mixing sequence, curing, and hardening procedure.

The mixing sequence was as follows:

− Mixing the dry powder with the activating solution at low speed for 10 min;
− Mixing the geopolymer paste with and without the acridine orange at high speed for 10 min. The mixture details are given in Table 2.

The GP composition was optimized in a previous study based on the following ratios: SiO_2_/Al_2_O_3_ = 4, Na_2_O/Al_2_O_3_ = 1, and H_2_O/Al_2_O_3_ = 13 [30]. The geopolymer synthesis was carried out with an AUCMA stand mixer SM-1815Z (AUCMA Co., Ltd., Qingdao, China). The fresh geopolymer pastes were placed into plastic molds and cured in an oven at a constant temperature of 25 °C for 24 h. After this curing, the samples were removed from the oven and aged at room temperature for 7, 14, 28, and 56 days. Geopolymer samples were grounded for 3 min at 90 rpm and with adjustable spring pressure by using a Retsch RM100 Mortar Grinder (Retsch GmbH, Haan, Germany). Before the analysis, the powdered samples were sieved at d < 125 µm. All the samples were analyzed at different ageing times. 

### 2.3. Geopolymer Characterization

#### 2.3.1. Chemical Stability

The chemical stability of the consolidated paste was checked with indirect measurements of the aluminosilicate 3D reticulation proposed in the literature [22,31]. Powders from the consolidated samples were immersed in water according to the procedure described in the Supplementary Materials, and the measurements of the pH and ionic conductivity of the eluates were indicators of the efficiency of the 3D reticulation. The higher the pH and ionic conductivity, the lower the networking degree. Additionally, pieces of the final geopolymers at different curing times were immersed in water, and the weight loss was used as an indicator of the reticulation over the 56 days after the preparation (see Supplementary Materials for details on the experimental procedures).

#### 2.3.2. FT-IR

Fourier-transform infrared spectroscopy (FT-IR) was performed by the Prestige21 Shimadzu system (Shimadzu, Milan, Italy). The instrument was equipped with a deuterated triglycine sulfate with potassium bromide windows (DTGS KBr) detector, with a resolution of 2 cm^−1^ and 60 scans. The analysis was carried out in the range of 400–4000 cm^−1^. The KBr disks were used for the analysis (2 mg of ground sample mixed with 198 mg of KBr). The FT-IR spectra were elaborated by IR solution (v.160, Shimadzu, Milan, Italy) and Origin (v.2022b, OriginLab Corporation, Northampton, MA, USA). The analyses were carried out on the samples aged 7, 14, 28, and 56 days at room temperature. 

#### 2.3.3. XRD

The X-ray diffraction patterns of the synthesized materials after 56 days of ageing were obtained using an X’Pert PRO X-ray diffractometer (Cu Kα1 radiation operated at 45 kV and 40 mA). X’Pert HighScore Plus (Malvern PANalytical software) was used for phase identification.

#### 2.3.4. SEM

Scanning electron microscopy (SEM) was used on Pt-coated samples (aged 56 days) using a Zeiss SIGMA field emission electron microscope operated at an accelerating voltage of 5 kV. 

#### 2.3.5. TGA/DTG

Thermogravimetric analysis (TGA) was conducted using a Precisa PrepASH 129 Thermogravimetric Analyzer. The samples were heated from room temperature to 1000 °C with a heating rate of 10 °C/min under N_2_ atmosphere. The specimens tested for weight loss in the TGA apparatus were the same powders used for the XRD analysis.

#### 2.3.6. Flexural Strength

**Figure 1:**
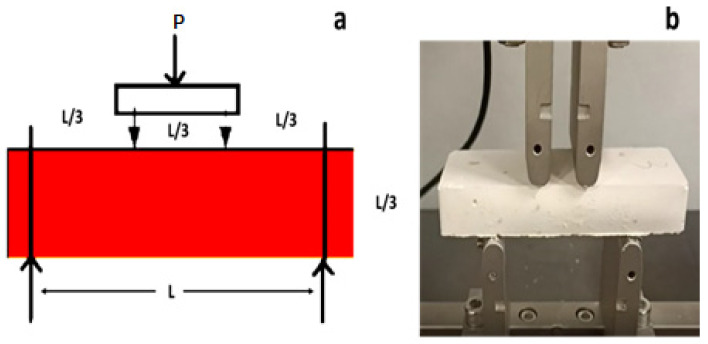
(**a**) Scheme of the four-point flexural test. (**b**) Flexural test experimental setup.

The mechanical behavior of the geopolymer systems was investigated by four-points flexural testing using a Dual Column Tabletop Testing System (INSTRON, series 5967-INSTRON, Norwood, MA, USA) configured with a crosshead speed of 1 MPa/min. The tests were conducted on five rectangular parallelepiped specimens (11 cm × 2 cm × 4 cm) with geometry according to ASTM C78 [32] or EN 12390-5 (EN 12390-5:2019—Testing hardened concrete—Part 5: Flexural strength of test specimens) [33] as shown in Figure 1a. The modulus of rupture is determined by the formula

(1) $$R=PL/bd^{2}$$

where *R* is the modulus of rupture, *P* is the maximum applied load indicated by the testing machine, *L* is the span length, and *b* and *d* are the average width and the depth of the specimen at the fracture, respectively. The configuration of the mechanical test is depicted in Figure 1b.

#### 2.3.7. Compressive Strength

The compressive strength (σ_max_) tests on five cubic (5 cm × 5 cm × 5 cm) specimens for each formulation were carried out by using an Instron 5567 electromechanical testing machine (maximum load 10 kN) at a constant displacement rate of 5 mm/min, according to European standard EN 826 [34]. 

#### 2.3.8. Antimicrobial Analysis

The antibacterial test was performed by the Kirby–Bauer method [35] on *S. aureus* (Gram-positive) and *E. coli* (Gram-negative) microbial strains. The whole adopted procedure can be described in five steps: (i) agar-based media preparation, (ii) sample preparation and sterilization, (iii) bacterial strain preparation, (iv) bacterial incubation, and (v) inhibition halo diameter (IHD) and bacterial viability (BV, %) measurements [36].

i. For media preparation, Tryptone Bile X-Gluc (TBX) Medium powder was dissolved in deionized water and autoclaved at 120 °C for 15 min. After cooling at 50 °C, the media was poured into Petri dishes, PD (6 cm in diameter), and stored at 4 °C before use. Baird–Parker Agar (BPA) powder was prepared following the same procedure as the TBX Medium. Before pouring into Petri dishes, the egg yolk supplement, containing potassium tellurite, was added to the BPA. Both bacterial media were purchased from Liofilchem, Roseto Degli Abruzzi, Italy.
ii. For sample preparation, 150 mg of MK, GP, and GPAO powders were pressed to obtain sample disks that were sterilized under UV light for 1 h.
iii. For bacterial strain preparation, *S. aureus* and *E. coli* bacterial strain pellets were dissolved in saline sterilized water (0.9% of NaCl), obtaining bacterial suspensions of 10^9^ CFU/mL. After the dissolution, *E. coli* was plated on TBX Medium, while the *S. aureus* was plated on BPA.
iv. For bacterial incubation, after bacterial plating, the sterilized samples were put in the centre of Petri dishes and incubated within the bacteria. *E. coli* was incubated at 44 °C for 24 h, while *S. aureus* was incubated at 36 °C for 24 h.
v. For *IHD* and *BV* measurement, four measurements of *IHD* were taken for each Petri dish to obtain both the mean and standard deviation. Bacterial viability (*BV* %) was calculated following Equation (2) as reported in [37]:

(2) BV=(PD−IHD)/PD∗100

#### 2.3.9. UV-Vis Analysis

Information about the presence and the amount of AO dye in GPAO after the alkali activation was obtained by the UV-Vis analysis. To this aim, the spectrum of AO (extracted in ethanol) was recorded with a Shimadzu UV-1800 UV-Visible Scanning Spectrophotometer (Shimadzu, Milan, Italy) in the range of 350–600 nm. The extraction procedure was divided into the following steps: (i) mix 1.00 g of GPAO powder with 25.0 mL of ethanol; (ii) shake for 10 min; (iii) centrifuge for 5 min at 1300 rpm; and (iv) recover and filter (0.45 μm) the supernatant. The spectrum was recorded after diluting 1:8 the filtered solution, and the amount of organic dye was determined. Moreover, also the amount of the organic dye released in the water (integrity test conditions, Supplementary Materials, Section S4) was quantified. Both the quantifications were carried out by recording the absorbance at λ = 490 nm, the maximum selected to build the calibration curve (Figure S5) as reported in [38]. 

## 3. Results

### 3.1. Sample Characterization

**Figure 2:**
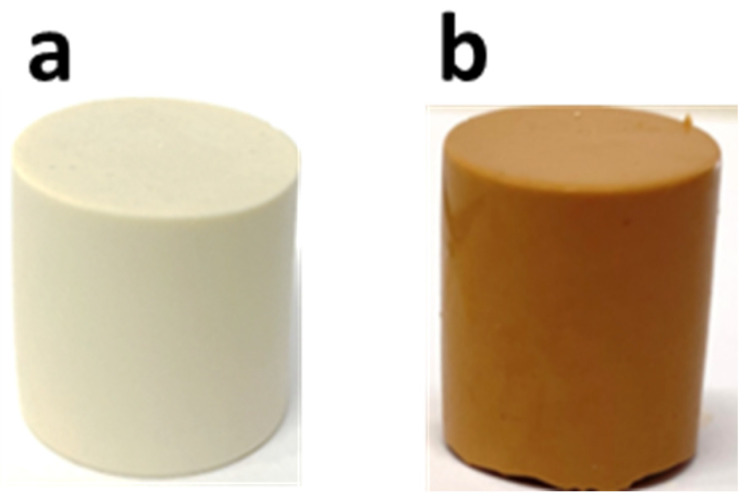
Images of the geopolymer specimen (**a**) without, GP, and (**b**) with acridine orange, GPAO.

Figure 2 shows the geopolymers obtained with and without the acridine orange dye. The images reveal that the organic dye was not degraded in the alkali environment of the geopolymeric paste. Moreover, both samples were homogeneous and showed no bubbles or cracks on their surfaces.

Ionic conductivity and pH measurements revealed that after 56 days of ageing time, the pH values were 12 (Figure S1), and the GPAO sample had an ionic conductivity of 500 mS/m (Figure S2B), which was higher than the one without the dye (300 mS/m, Figure S2A). Both samples were completely hardened after 56 days of ageing time; indeed, they did not break after the integrity test (Figure S3 and Table S1) and had a weight loss lower than 1.5% after 56 days of ageing (Figure S4). GPAO released a small amount of the dye into the eluate water after both the integrity and weight loss tests.

These first tests indicated that the geopolymer reticulation occurred regularly even in the presence of AO. The pH and the ionic conductivity values of the eluates are in the range of almost completely reacted MK geopolymers [30,39]. The aluminosilicate network retained the dye, which was only slightly released in water also because of its insolubility in aqueous solutions.

### 3.2. FT-IR Characterization

**Figure 3:**
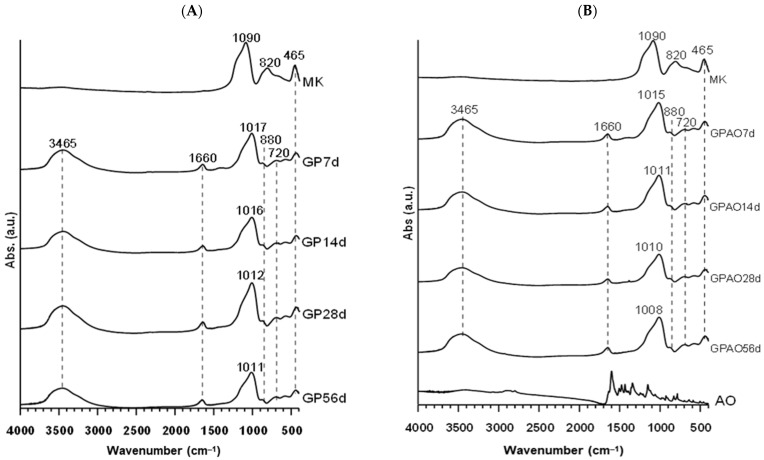
(**A**) FT-IR spectra of MK and GP at different ageing times. (**B**) FT-IR spectra of MK, AO, and GPAO at different ageing times.

The geopolymerization process was evaluated by determining the DOPSM (density of state of peak maximum) shift of the band at 1090 cm^−1^ of MK [22,40,41]. Indeed, as the geopolymerization occurred, the band at 1090 cm^−1^ assigned to the asymmetric stretching of Si-O-T (T = Si or Al) shifted at 1017 (7 days) to 1011 cm^−1^ (58 days, Figure 3A) in the GP sample. This shift has been already explained in the literature by the substitution of Si by Al atoms in the 3D network [36,40,41]. The bands at 820 cm^−1^ and 880 cm^−1^ were assigned to Al(IV)-OH and Al(IV)-O- vibrations, whereas the signal at 465 cm^−1^ was assigned to Si-OH symmetric bending [42,43]. In addition, the band at 3645 cm^−1^ and a weak band at 1645 cm^−1^ were assigned to the -OH stretching and bending deriving from both water and silanol molecules. Furthermore, FT-IR spectroscopic results indicated that the presence of the organic dye did not affect the geopolymerization occurrence of the GPAO samples. Indeed, there was the DOSPM shift from 1090 to 1008 cm^−1^ (56 days) in the GPAO sample spectra (Figure 3B).

**Figure 4:**
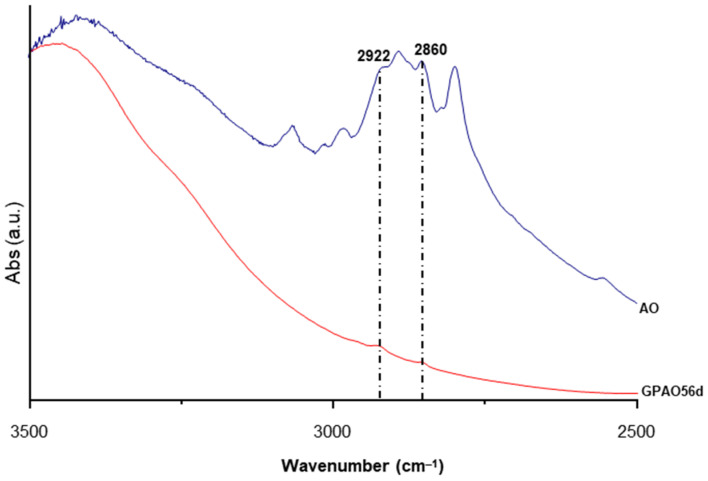
FT-IR spectrum of AO and GPAO aged up to 56 days.

Given the small amount of the organic dye, no strong signals were appreciable in the GPAO IR spectra. However, an enlargement of the range 3000–2500 cm^−1^ revealed the presence of weak signals related to the vibration of -CH_2_ [44,45,46] (Figure 4).

The encapsulation of AO in geopolymeric pastes did not alter the regular consolidation process in terms of aluminosilicate network creation starting from the metakaolin disordered structure. The large bands of the FT-IR spectra collected on GP and GPAO samples indicated a similarly amorphous aluminosilicate structure where the bands at 1090 and 820 cm^−1^ of MK were substituted by a single 1011–1008 cm^−1^ band in 56 days at room temperature. The shift of the 1090 cm^−1^ band occurred in the first 7 days of curing, while in the remaining 56 days, a very slight additional shift was recorded in the direction of Al incorporation in the silicate structure.

### 3.3. XRD 

**Figure 5:**
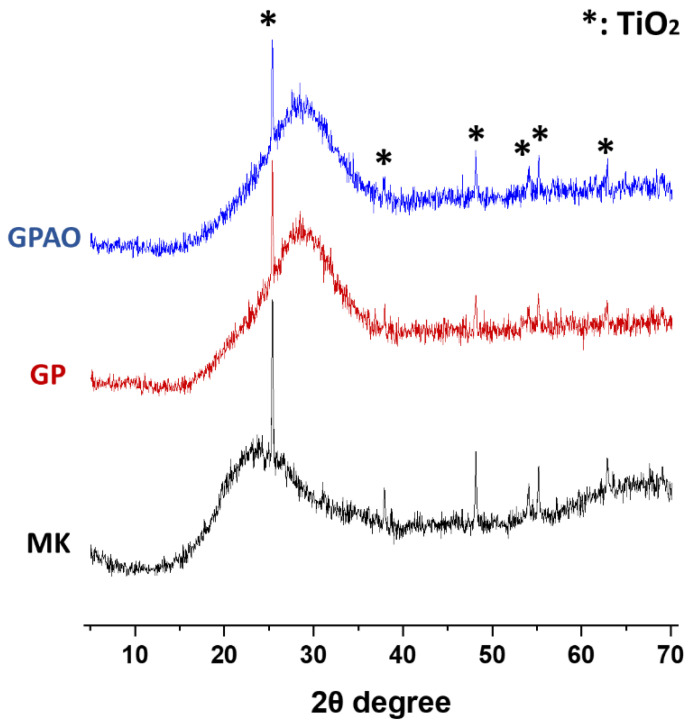
XRD diffraction patterns of the used MK and the prepared GP and GPAO aged 56 days.

The XRD diffraction patterns of the metakaolin and prepared geopolymers are shown in Figure 5. MK displayed an amorphous feature between 15 and 35° 2θ with sharp reflections arising from crystalline TiO_2_ present as impurities in MK. The formed geopolymer (GP) displayed a similar amorphous hump shifted to the range 20–40° 2θ, typical for a “well-formed” geopolymer [47,48,49], on which were superimposed the sharp reflections ascribed to the TiO_2_ impurities. The geopolymer with acridine orange (GPAO) displayed a similar XRD pattern to that of GP, indicating that the presence of the organic dye did not result in any mineralogical changes to the formed geopolymer and thus did not interfere with the geopolymerization process.

The XRD diffraction patterns confirmed the amorphous nature of the geopolymeric matrices formed in the GP and GPAO samples. Additionally, the shift toward higher 2θ angles of the amorphous halo confirmed the insertion of Al in the silicate network, typical of the geopolymerization process [50].

### 3.4. SEM 

**Figure 6:**
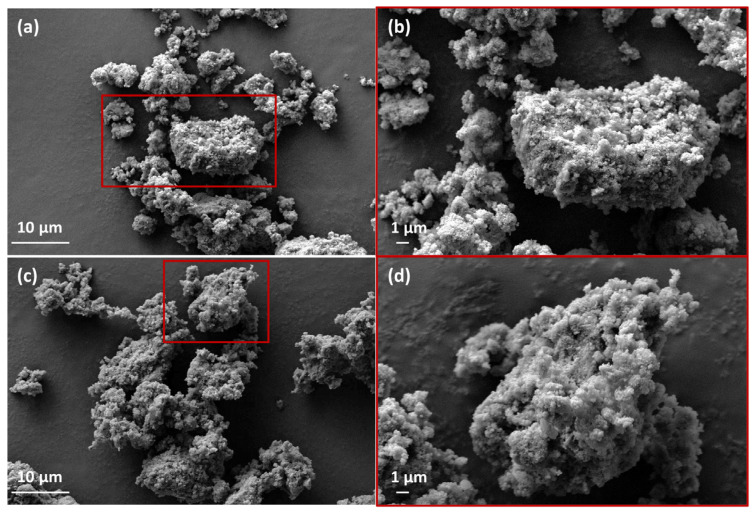
SEM micrographs at different magnifications for the prepared geopolymers, GP (**a**,**b**) and GPAO (**c**,**d**).

The morphology of the prepared geopolymers without (Figure 6a,b) and with acridine orange organic dye (Figure 6c,d) was investigated by SEM as shown in Figure 6. Both geopolymers showed similar amorphous randomly shaped particles composed of smaller particles aggregating to form voids within larger particles.

The obtained SEM images (Figure 6) show no differences in the morphological characteristics of the prepared geopolymers (with or without the organic dye). This indicates the suitability of geopolymers in applications such as the encapsulation of organic pollutants or dyes without influencing their structural stability and characteristics.

### 3.5. TGA/DTG

**Figure 7:**
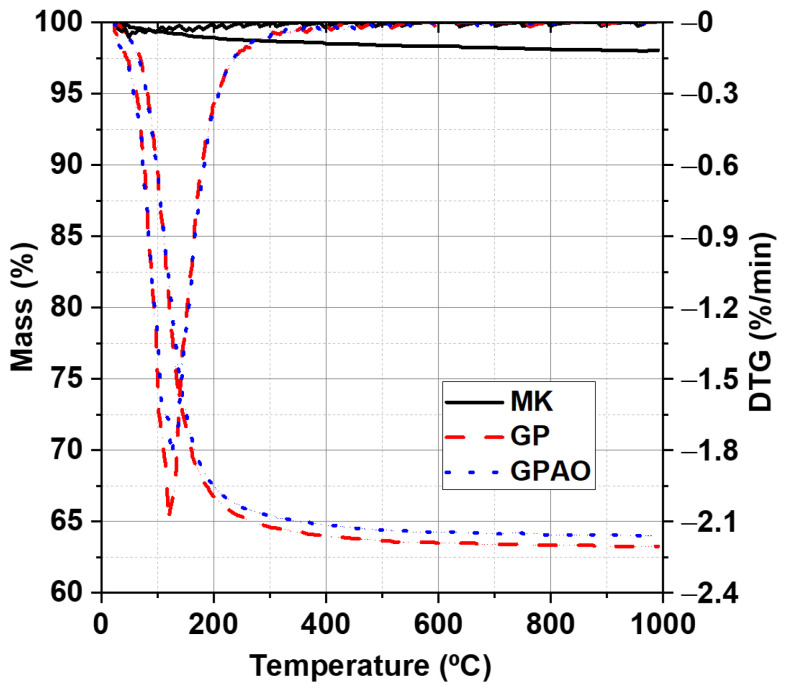
TGA/DTG profiles for the MK and the prepared GP and GPAO.

The TGA/DTG profiles for the MK and the prepared geopolymers are shown in Figure 7. MK displayed a slight weight loss (~2.5 wt.%) at temp < 300 °C, which could be ascribed to the removal of adsorbed atmospheric moisture. The formed geopolymers, however, showed a significant weight loss (up to 37.5 wt% over a total water content of 39.3 and 39.2 wt% calculated for GP and GPAO fresh paste, respectively) between 50 and 350 °C due to dehydration, which is typical for geopolymer materials [51]. As shown in Figure 7, geopolymers both with acridine orange dye (GPAO) and without (GP) displayed almost similar TGA profiles with a negligible difference. The desorption of the organic dye was not detectable via TGA most probably due to the low amount of dye added to the geopolymer mixture (Table 2).

### 3.6. Flexural Strength

The determination of the flexural strength of a material (defined as the maximum amount of tensile load to which a material can be subjected before failure) is an important property for identifying the fields of application of the material because it is related to its structural integrity, strength, and performance. The analyzed sample showed an average maximum flexural strength of 6.5 ± 0.8 MPa. The introduction of acridine orange dye did not substantially change the structure of the geopolymer, as a value of 5.8 ± 1.1 MPa was found. These results were in accordance with those found in [52,53], which reported flexural strength of 6 MPa after 28 days of ageing time for geopolymer systems with a water–solid ratio equal to 0.36. Moreover, many papers also report on the lowering of flexural strength properties with the entrapment of wastes inside metakaolin-based geopolymers [52,54,55].

Although the composition of the material plays a significant role in the correct use of technology of geopolymers, the addition to the aluminosilicate source material of reinforcing elements or elements of another nature can change the mechanical characteristics of the hardened material. In our case, the flexural performance of GP and GPAO-dense hardened materials was to be considered good if compared with other studies in which admixed geopolymers were produced. The value 6.5 MPa was a medium-high value that fell in the range of flexural strength of many admixed systems that present elements such as silica fume, steel fibers, nano-silica, etc. [56]. 

The same considerations could be extended to the compressive strength values obtained for GP at approximately 22.52 MPa and 1% lower for GPAO (with 20.57 MPa) calculated as average on five cubic specimens. The slight decrease in mechanical strength could be related to the presence of ethanol in the paste, which might have generated some pores during evaporation from the viscous fresh paste.

### 3.7. Antimicrobial Analysis

**Figure 8:**
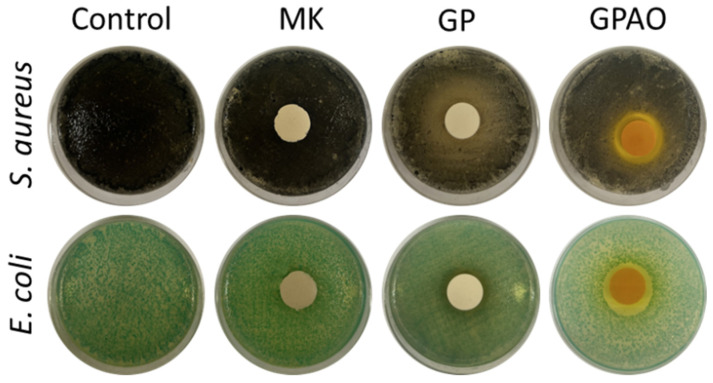
Sample images after the incubation with the bacterial strains.

Aiming to evaluate the possible application field of the consolidated geopolymers (such as decorative home objects), the bioimpact of GP and GPAO was assayed in the presence of *S. aureus* and *E. coli* (Figure 8), and both the IHD and BV (%) were evaluated. 

**Figure 9:**
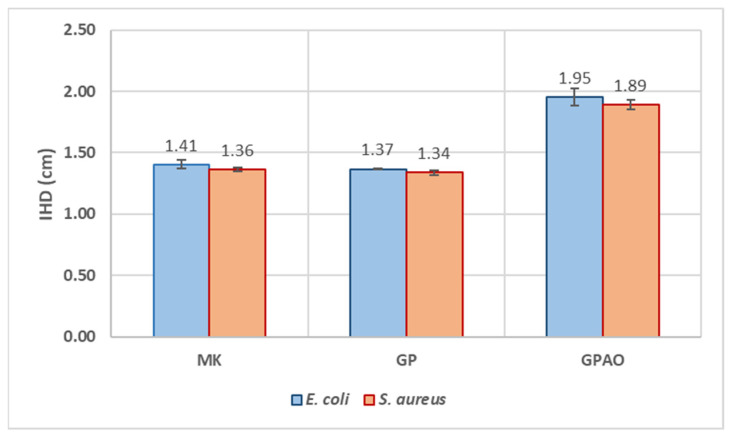
IHD values of MK, GP, and GPAO assayed in the presence of *E. coli* and *S. aureus*.

**Figure 10:**
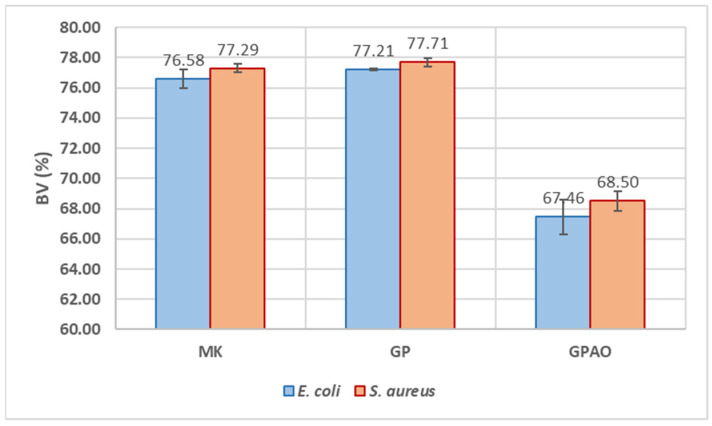
BV (%) of MK, GP, and GPAO assayed in the presence of *E. coli* and *S. aureus*.

Figure 9 shows the results of the IHD measurements. Growths of *E. coli* and *S. aureus* were not detected on MK and GP samples, which seemed to be inert against these bacteria. However, the presence of acridine orange increased antimicrobial activity. Indeed, the IHD values were 1.94 ± 0.07 cm in the presence of *E. coli* and 1.89 ± 0.04 cm in regard to *S. aureus*. As a consequence, also the BV (%) was highly decreased (BV = 67.5 ± 1.2% and 68.5 ± 0.7%) as shown in Figure 10. The antimicrobial effect of acridine orange was also in accordance with the literature [57].

These results indicated that the alkaline environment typical of the geopolymeric materials and measured in water as pH = 12 was not affecting the proliferation of the two bacterial colonies, while the presence of acridine orange was significantly inhibiting their growth. The alkaline environment of the geopolymers did not affect the antibacterial properties specific to the macromolecules of the dye, which retained its typical reactivity (as shown by the FT-IR results), as it retained the color (see comments in Section 3.1).

### 3.8. UV-Vis Analysis and Release Study

**Figure 11:**
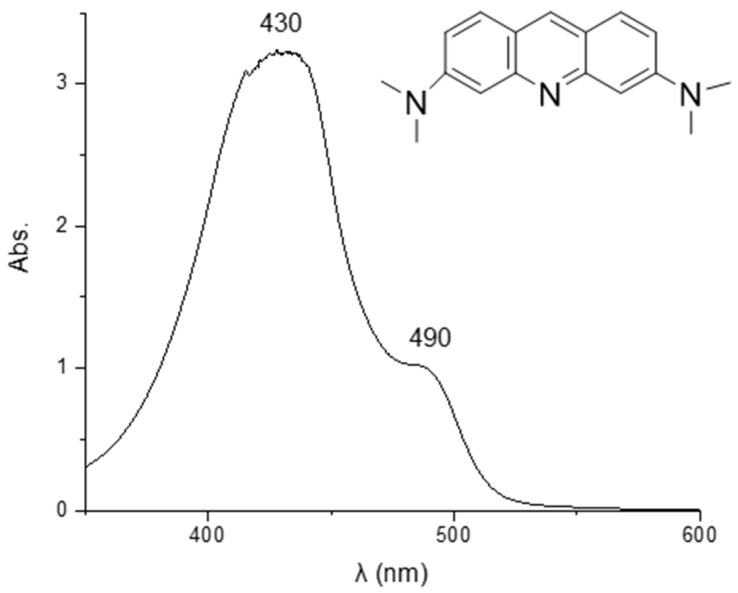
UV-Vis spectrum of acridine orange in ethanol (solution diluted 1:8).

The presence of the organic dye in GPAO was determined by UV-Vis analysis. The spectrum of AO extracted in ethanol from GPAO in the range of 350–600 nm (Figure 11) reported one main peak at 430 nm with a shoulder at 490 nm. The former was due to the aggregate formation of the dye inside the alcoholic solution, whereas the latter was related to the monomeric form of the acridine orange. Both the signals (aggregate and monomeric forms) were due to the π–π* transition of the conjugated rings [58,59]. According to the calibration curve (Figure S5), the amount of the organic dye extracted from GPAO was 2.48 mg per 1.00 g of GPAO, which was close to the theoretical amount (2.97 mg per 1.00 g of GPAO) belonging to the geopolymer formulation. 

Regarding the release study in water, the results revealed that after 8 and 24 h of release, the absorbance values of the solution (recorded at λ = 490 nm) were 0.071 and 0.064, respectively. These values were lower than the absorbance of 0.111 recorded for the concentration of 2 µg/mL (that is the LOD of the built calibration curve, Figure S5), suggesting the high retention of the organic dye within the geopolymer matrix.

## 4. Discussion

Many studies have reported the ability of geopolymers to entrap solid [8,60] and liquid [61] wastes inside their 3D structures, proposing new eco-friendly solutions and giving an added value to the wastes without their disposal in the environment. Some authors have focused their research on the entrapment of hazardous macromolecules and organic dyes [62,63]. Following this trend, in our previous study, the feasibility to synthesize metakaolin-based geopolymers cured at 25 and 40 °C in the presence of pH indicators (phenolphtalein, cresol red, methyl orange, and bromothymol blue) [22] was demonstrated. The direct entrapment of these molecules into the fresh paste of geopolymers did not alter the normal geopolymerization occurrences and obtained colored materials with possible applications also for decoration. However, the formulation and syntheses proposed did not lead to materials able to retain their colors once soaked in water for a long time. This finding was in accordance with MacKenzie and O’Leary [64], which tried to synthesize geopolymers with other acid–base indicators as tools to reveal color-change humidity. Contrary to our previous study, the GPAO demonstrated a good geopolymerization and retaining of the dye that was not released in water. In addition to this feature, the presence of titania (shown in the XRD spectrum) and the ability to inhibit microbial growth could represent good properties for the application of this geopolymer in the restoration field [65] and building façade self-cleaning [66,67] and could contribute to the research in hazardous dye inertization. 

## 5. Conclusions

In this paper, the feasibility to obtain colored geopolymers with the entrapment of acridine orange was investigated. In particular:

- FT-IR revealed the presence of the AO in GPAO and the occurrence of geopolymerization (DOSPM shift at lower wavenumbers), supported also by the XRD analysis (see the amorphous hump shift to the range 20–40° 2θ).
- The physical-chemical properties (analyzed through pH and IC, and weight loss and integrity tests) of the samples revealed no huge differences between GP and GPAO. These indirect data on the stability of both samples were also strengthened by the TGA/DTG and SEM analyses.
- The slight decrease in mechanical and flexural strengths of GPAO with respect to GP could be explained by the formation of some pores during solvent evaporation from the viscous fresh paste.
- The UV-Vis spectrum of AO extracted from GPAO supported that the alkaline environment required for the geopolymerization did not degrade the organic dye, which was also retained without being released (concentration of AO released in water lower than the LOD of titration curve).
- The investigation of the antimicrobial activity of GP and GPAO revealed increased activity of the specimens with acridine orange against *E. coli* and *S. aureus* bacterial strains probably due to the very low amount of dye release.

Even if all these results are promising, further investigations are needed to evaluate the applicability of the above-formulated geopolymer as a material for restoration or for catalytic and eventually self-cleaning applications in building façades, because of the TiO_2_ in the white metakaolin, and for toxic dye inertization by directly using wastewaters.

## Figures and Tables

**Table 1:** Chemical composition of the materials.

Compound (wt%)	SiO_2_	Al_2_O_3_	TiO_2_	Na_2_O	Other Oxides	H_2_O
White metakaolin	53 ^1^	40.5 ^1^	5 ^1^	-	1.5 ^1^	-
Sodium silicate solution	27.1	-	-	8.85	-	64.05

^1^ Average value adapted from [22].

**Table 2:** Mixture details of geopolymers with and without the organic dye.

Sample Name	MK	Activator Solution	Liquid/Solid Ratio	AO
GP	50 g	79.4 g	0.36	-
GPAO	50 g	79.4 g	0.36	0.4 g (in 6 mL of ethanol)

## Data Availability

The data presented in this study are available on request from the corresponding authors.

## Supplementary Materials

The following supporting information can be downloaded at https://www.mdpi.com/article/10.3390/polym15030675/s1: Figure S1: pH measurements of (A) GP and (B) GPA at different ageing times; Figure S2: IC measurements of (A) GP and (B) GPA at different ageing times; Figure S3: Geopolymer samples after the integrity test; Figure S4: Weight loss results of the geopolymer samples at different ageing times; Figure S5: Calibration curve of AO in water; Table S1: Results of the integrity test.
