# Supplementary material for: Entrapment of Acridine Orange in Metakaolin-Based Geopolymer: A Feasibility Study
Source: Polymers (Basel). 2023 Jan 28;15(3):675. doi: 10.3390/polym15030675 (PMC9919871; doi:10.3390/polym15030675)
Supplement: Supplementary file 1 [file polymers-15-00675-s001.zip › polymers-2169504-supplementary.pdf]

Supplementary Material

Entrapment of Acridine Orange in Metakaolin-Based Geopolymer: A Feasibility Study

1. Ionic conductivity and pH

Ionic conductivity (IC) and pH were measured by dissolving the geopolymer samples (crushed and sieved (d < 0,125 μm)) in deionized water (1/10 w/v) in accordance with [1]. The IC and pH values were collected at different times (t_1 = 0 min, t_2 = 5 min, t_3 = 10 min, t_4 = 30 min, t_5 = 1 h, t_6 = 2 h, t_7 = 4 h, t_8 = 6 h, t_9 = 12 h, t_{10} = 24 h, t_{11} = 48 h, and t_{12} = 72 h) for all aged samples. The IC measurements were carried out with a Crison GLP31 (conductivity cell 50 72, made up of glass and platinum, measuring range from 0 to 50,000 μS/cm, and temperature range from −35 to 85 °C), whereas the pH measurements were performed with a Crison GLP21 (pH measuring range 0–14, reference element of Ag/AgCl with Ag^+ ion barrier, and operating temperature range −10 to 100 °C). Both instruments were made by Hach Lange S.L.U., Barcelona, Spain. Three independent measurements were done to measure the standard deviation.

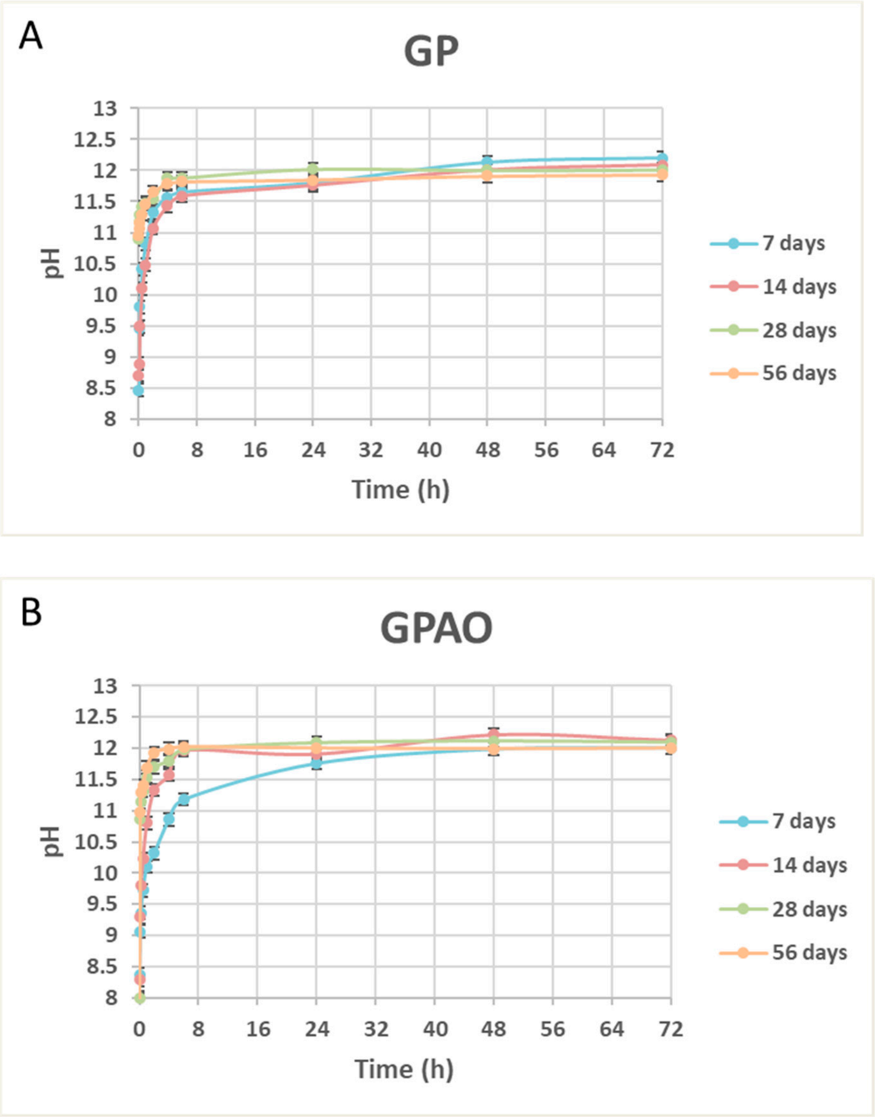

Figure S1. pH measurements of (A) GP and (B) GPA at different ageing times.

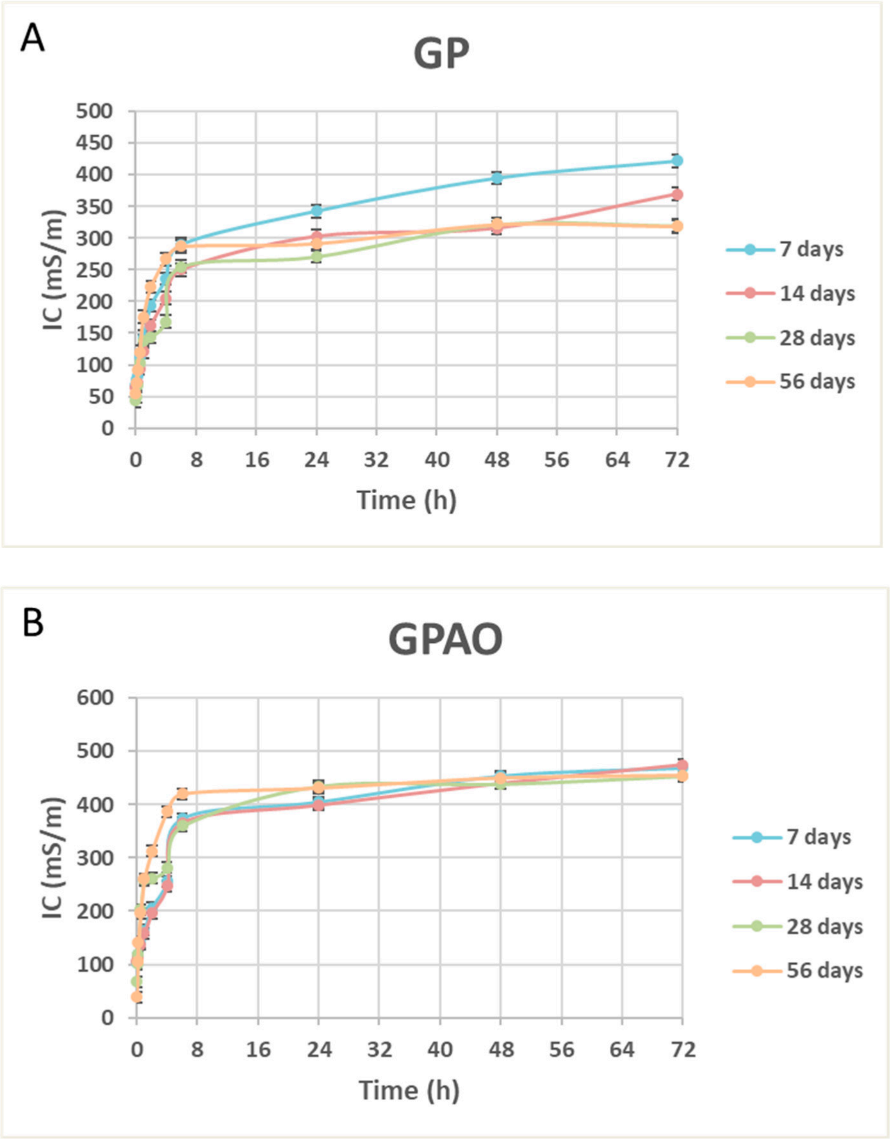

Figure S2. IC measurements of (A) GP and (B) GPA at different ageing times.

2. Integrity test

The integrity test was performed by soaking the geopolymers in MilliQ water (1/100 w/v) for 24 h. After 24 h , the sample integrity was estimated adopting the procedure reported in [1,2].

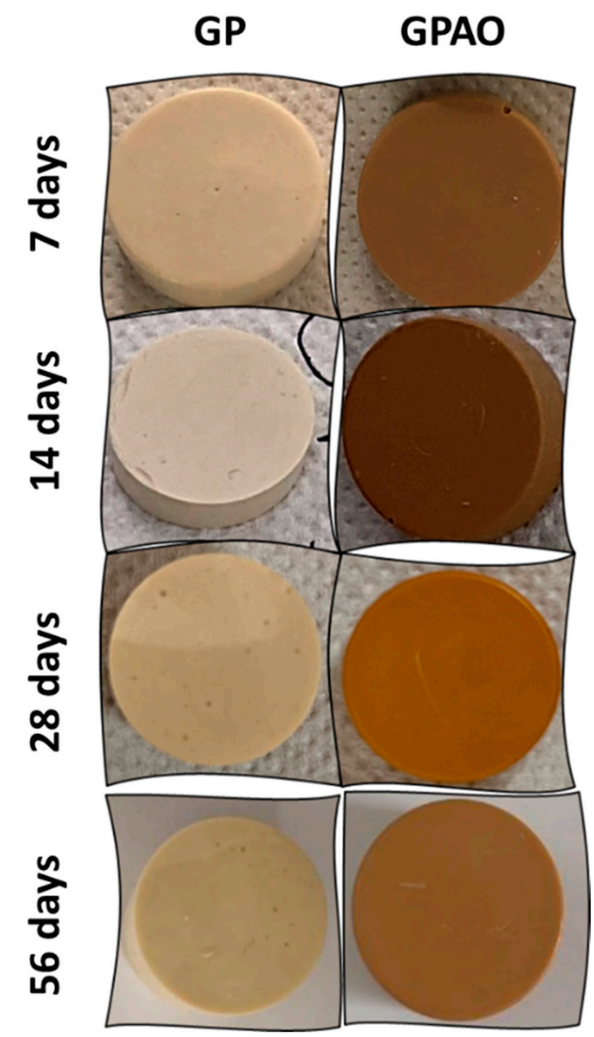

Figure S3. Geopolymer samples after the integrity tests.

Table S1. Results of the integrity test.

	**Time (days)**	**7**	**14**	**28**	**56**
GP	**pH**	9.80	9.04	9.55	9.81
	**Integrity**	No	No	Yes	Yes
	**Water properties**	Clear	Clear	Clear	Clear
GPAO	**pH**	10.33	8.00	9.88	9.14
	**Integrity**	No	Yes	Yes	Yes
	**Water properties**	Clear, light yellow	Clear, Light yellow	Clear, light yellow	Clear, light yellow

3. Weight loss test

The samples were broken into large pieces, dried for 3 h in acetone and then in an oven at 25°C, and finally weighed (Wi) and soaked in Milli-Q water (1:100 w:v) for 24 h. After 24 h, the pieces were removed from the water and immersed for 3 h in acetone. After drying in the oven at 25°C for 3 h, the pieces were weighed (Wf), and the percentage of weight loss (WL) was calculated following equation 1 [2]:

$$WL = \frac{Wi - Wf}{Wi} * 100 \quad (1)$$

Three independent measurements were taken to measure the standard deviation.

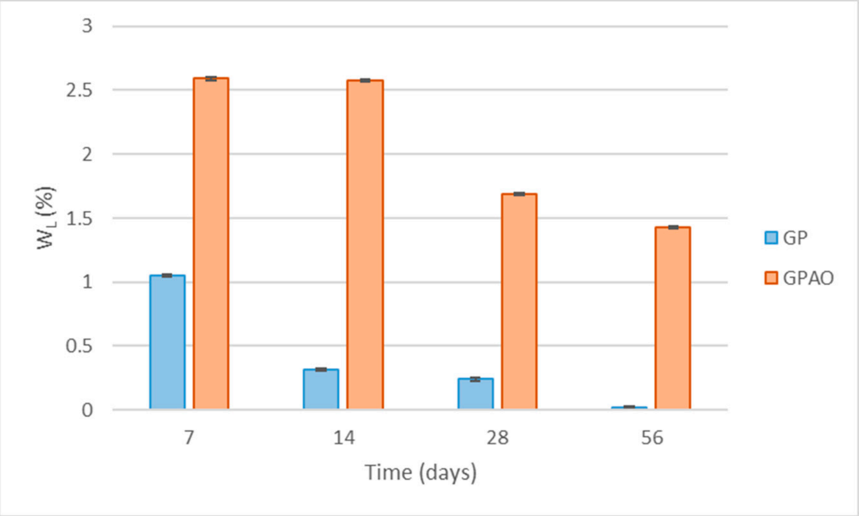

Figure S4. Weight loss results of the geopolymer samples at different ageing times.

4. UV/Vis analysis

A Shimadzu UV-1800 UV/Visible Scanning Spectrophotometer (Shimadzu, Milan, Italy) was used to build the calibration curve (Figure S5), recording the absorbance at λ = 490 nm in the range of 0-50 µg/mL (acridine orange dissolved in water) [3], with R^2 = 0.9952. The limit of detection (LOD) was taken at 2 µg/mL, whereas the limit of quantification (LOQ) was calculated at 6.6 µg/mL.

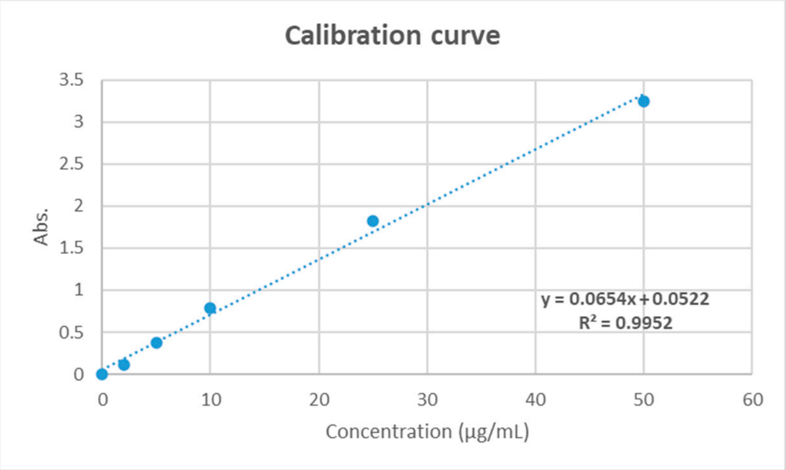

Figure S5. Calibration curve of AO in water.

References

1. Sgarlata, C.; Formia, A.; Ferrari, F.; Leonelli, C. Effect of the Introduction of Reactive Fillers and Metakaolin in Waste Clay-Based Materials for Geopolymerization Processes. *Molecules* **2021**, *26*, 1325. https://doi.org/10.3390/molecules26051325.

2. D’Angelo, A.; Dal Poggetto, G.; Piccolella, S.; Leonelli, C.; Catauro, M. Characterisation of White Metakaolin-Based Geopolymers Doped with Synthetic Organic Dyes. *Polymers* **2022**, *14*, 3380. https://doi.org/10.3390/polym14163380.
3. Fiallos, D.C.; Gómez, C.V.; Usca, G.T., Pérez, D.C.; Tavolaro, P.; Martino, G.; Caputi, L.S.; Tavolaro, A. Removal of Acridine Orange from Water by Graphene Oxide. *AIP Conference Proceedings* **2015**, *1646*, 79. https://doi.org/10.1063/1.4908586
